# Understanding and Targeting Apoptotic Pathways in Ovarian Cancer

**DOI:** 10.3390/cancers11111631

**Published:** 2019-10-24

**Authors:** Linah F. Al-Alem, Andrew T. Baker, Unnati M. Pandya, Eric L. Eisenhauer, Bo R. Rueda

**Affiliations:** 1 Vincent Center for Reproductive Biology, Department of Obstetrics and Gynecology, Massachusetts General Hospital, Boston, MA 02114, USA; lal-alem@mgh.harvard.edu (L.F.A.-A.); abaker0096@gmail.com (A.T.B.); UPANDYA@mgh.harvard.edu (U.M.P.); Eric.Eisenhauer@MGH.HARVARD.EDU (E.L.E.); 2 Obstetrics and Gynecology, Harvard Medical School, Boston, MA 02115, USA; 3 Gynecology and Oncology Division, Department of Obstetrics and Gynecology, Massachusetts General Hospital, Boston, MA 02114, USA

**Keywords:** apoptosis, ovarian cancer, chemoresistance, glycosylation, miRNA, clinical trials

## Abstract

Ovarian cancer cells evade the immune system as well as chemotherapeutic and/or biologic treatments through inherent or acquired mechanisms of survival and drug resistance. Depending on the cell type and the stimuli, this threshold can range from external forces such as blunt trauma to programmed processes such as apoptosis, autophagy, or necroptosis. This review focuses on apoptosis, which is one form of programmed cell death. It highlights the multiple signaling pathways that promote or inhibit apoptosis and reviews current clinical therapies that target apoptotic pathways in ovarian cancer.

## 1. Introduction

Ovarian cancer (OvCa) is the fifth leading cause of cancer-related deaths in women in the United States [1]. Moreover, it is the leading cause of death from gynecologic malignancies and despite recent advances in research and treatment strategies, most patients still die from their disease [1]. Similar to most solid tumor cells, malignant ovarian cells depend on inherent or acquired mechanisms to escape cell death. Understanding the processes involved in cell death has become significantly more complex over the past several years and many forms of cell death have been characterized. This review focuses on one form of a programmed cell death process known as apoptosis. It will provide a general background on the different types of cell death and how they may differ from apoptosis. We discuss how evading apoptosis contributes to chemoresistance, and survival of the tumor cells. Lastly, we review current treatment strategies and pre-clinical studies that are designed to target and override anti-apoptotic factors in OvCa. 

### 1.1. Historical View of Cell Death

**Figure 1:**
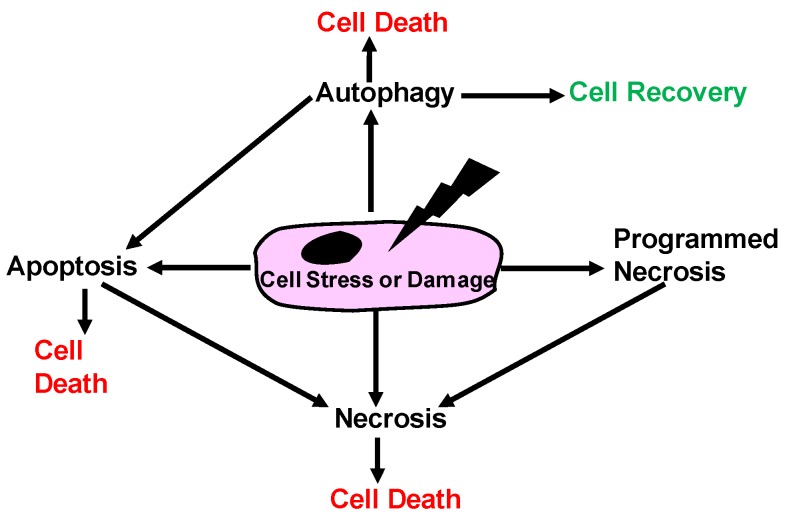
Cell death mechanisms: A cellular damaging or stressful event could push the cell toward recovery or one of the different cell death pathways including necrosis, apoptosis and autophagy.

Different processes of cell death have been described extensively [2]. Briefly, cell death can occur via necrosis, necroptosis, autophagy, apoptosis, or the cell can remain in a state of senescence [2,3]. Historically, the senescence model was refined by Hayflick and Morehead in 1961 when they established that human fibroblasts could divide in culture, and then cease replication after 40–50 cycles [4]. Shortly thereafter, Christian de Duve described autophagy in 1963 as a form of controlled cell death [5]. The apoptotic form of cell death was further characterized as a programmed cellular event until 1965 by Lockshin and Williams [6]. Finally, necrosis was differentiated from apoptosis via morphological observations in 1972 by Kerr and colleagues [7] as a non-programmed pathological form of cell death and apoptosis as a programmed form of cell death. Furthermore, scientists investigated a subset of cells where the apoptotic pathway was inhibited, and the cell displayed morphological changes that were consistent with both apoptosis and necrosis. This ‘programmed’ necrosis was then called necroptosis. [8]. Other types of cell death have since been described and include parthanosis, ferroptosis, and pyrotosis [2]. Figure 1 depicts a schematic of some of the various paths through which a cell dies.

### 1.2. Apoptosis 

**Figure 2:**
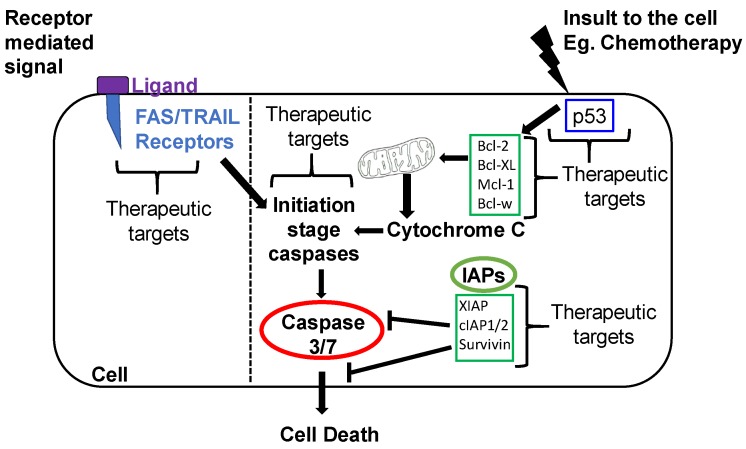
Simplified overview of extrinsic and intrinsic apoptosis pathways and inhibitor of apoptosis (IAP) function with drug targets.

Apoptosis occurs through one of two mechanisms (Figure 2): an extrinsic pathway (initiated outside the cell) that is receptor-dependent, or an intrinsic pathway that is mitochondria-dependent [9]. 

#### 1.2.1. Intrinsic Apoptotic Pathway

**Figure 3:**
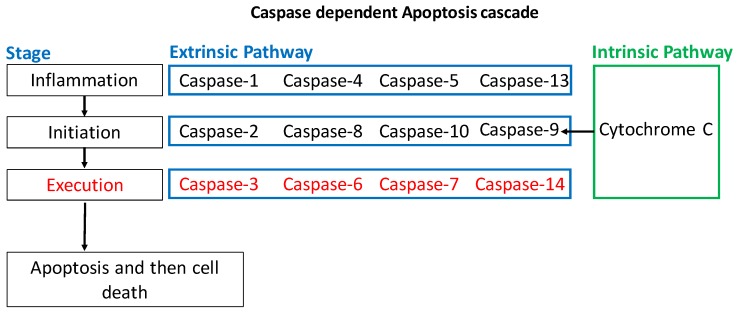
A summary of caspases identified and their major roles in the apoptotic cascade. The caspases identified under the execution stage (red) are typically the more heavily studied in cancer due to their presumed direct effect on apoptosis. The intrinsic apoptotic pathway culminates in the release of Cytochrome C, which then activates initiating caspases (e.g., Caspase 9. This activation then leads to apoptosis via execution caspases (e.g., Caspase-3).

The presence of an insult to the cells causes a cascade of events intracellularly that culminate in apoptosis. There are several proteins involved in the regulation of the intrinsic pathway, such as the Bcl-2 family. The Bcl-2 family has both pro- and anti-apoptotic members. The pro-apoptotic Bcl-2 family members are Bax, Bak, Bok, Bid, Bim, Bik, Bad, Noxa, and Puma [10]. Anti-apoptotic members include Bcl-2 and Bcl-XL. They inhibit apoptosis by blocking the release of Cytochrome C from the mitochondria. The release of Cytochrome C in turn activates Caspase-9, which then goes on to activate Caspase-3 and Caspase-7 (Figure 3). Other anti-apoptotic members include A1, Bcl-w, and Mcl1 [10]. An imbalance in the ratio of pro-apoptotic to anti-apoptotic players can tip the scales in one direction or another. For instance, Bad functions by inactivating the anti-apoptotic members of the Bcl-2 family (Bcl-2, Bcl-xL, Bcl-w) which leads to the inhibition of cell survival and increased apoptosis [11]. Bax and Bak are also able to stimulate the release of Cytochrome C from mitochondria leading to apoptosis [9]. Their importance was demonstrated by showing that cells become resistant to apoptosis stimuli if the genes encoding Bax and Bak are both inactivated [9]. Conversely, Bax and Bak are activated by other apoptosis-promoting members of the Bcl-2 family such as Bid. In general, apoptosis relies heavily on a family of procaspases are activated via adapter proteins. Once cleaved, their active form induces a cascade of proteolytic activity, leading to apoptosis [9]. Caspase-mediated programmed cell death has been studied extensively, and is a primary conduit involved in the apoptotic process (Figure 2). Other pro-apoptotic proteins such as Noxa and Puma increase in response to DNA breaks and chromosomal abnormalities. This increase is typically mediated via TP53 whose loss of function is common in cancer cells. In the absence of a functional TP53, many cells escape apoptosis [12]. Similar to many cancers, p53 in OvCa is mutated in more than half of the cases [13]. 

**Figure 4:**
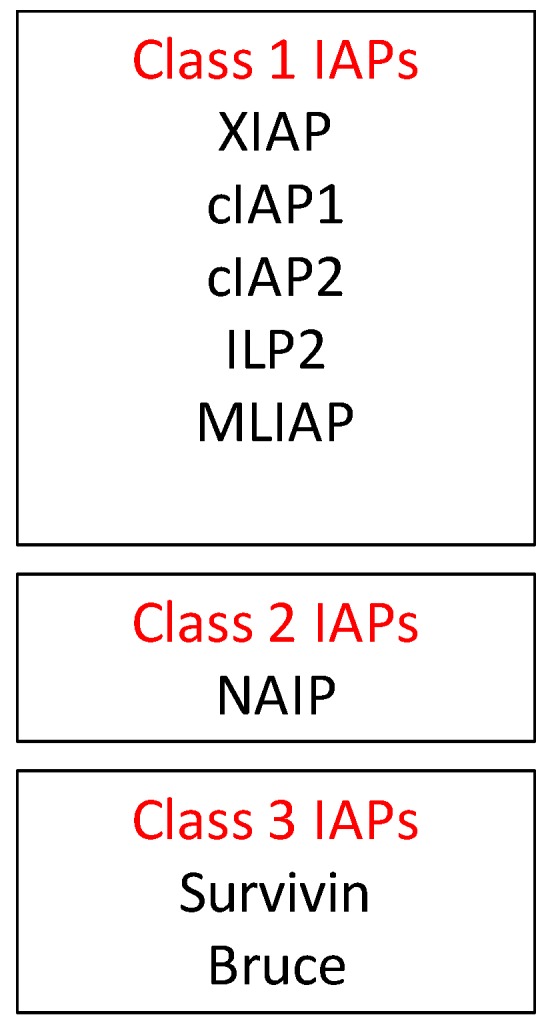
Proteins under each class of IAPs.

Another family of proteins regulating intracellular apoptosis is the IAP (inhibitor of apoptosis) family. In mammals, there are eight known IAPs grouped into three classes shown in Figure 4 [14,15]. These inhibitors can bind to specific procaspases and prevent their activation. IAPs are frequently upregulated in cancer and promote tumor maintenance and/or progression. IAPs are known to activate major cell signaling pathways involving key regulators such as nuclear factor κB (NF-κB) and mitogen-activated protein kinase (MAPK) and can drive the expression of genes that are important in inflammation, immunity, and cell survival. IAPs play a significant role in regulating apoptotic signals from the intrinsic as well as extrinsic apoptotic pathways. 

#### 1.2.2. Extrinsic Apoptotic Pathway

In the extrinsic pathway of apoptosis, specific ligands induce a cascade of events inside the cell that culminates in apoptosis as illustrated in Figure 1. This pathway is sometimes referred to as the receptor-mediated or death receptor pathway and includes Fas receptor, tumor necrosis factor (TNF) receptor, and TNF-related apoptosis-inducing ligand (TRAIL) receptor [14]. Specific ligands such as TRAIL or Fas ligand (FasL) binding to TRAIL or Fas receptors, respectively trigger the aggregation of their respective receptors on the target cell [2]. After aggregation, adaptor proteins known as Fas-associated death domain protein (FADD) are recruited on the cytoplasmic side of the receptors. FADD, in turn, then recruits Caspase-8, which is an initiator protein, to form the death-inducing signal complex (DISC). In this complex, Caspase-8 is activated by being cleaved from its full-length form (p55/53) to p43/41 and p10 fragments. These fragments can then cleave pro-Caspase-3 into the active Caspase-3, thereby initiating cell degradation. Active Caspase-8 can also cleave the BID protein to tBID, which acts as a signal on the membrane of mitochondria to facilitate the release of Cytochrome C in the intrinsic pathway. More specific details of the different members involved in the extrinsic apoptotic pathway can be found in the literature [3]. 

## 2. Apoptotic Mechanisms Altered in OvCa

### 2.1. Activators of Apoptosis in OvCa

Direct changes in levels of pro-apoptotic proteins have been the focus of some studies in OvCa, especially in the context of chemoresistance and chemosensitivity. Yang et al. using multicellular spheroids from OVCAR3 and SKOV3 OvCa cell lines showed that suspended and adherent cells were more susceptible to death when treated with cisplatin compared to multicellular spheroids [16]. The authors determined that the protein level of Bcl-2 was higher in multicellular spheroids compared to adherent cells in the presence of cisplatin in SKOV-3 and OVCAR3 cell lines. They also showed there was a lower level of Caspase-3 and Caspase-9 in multicellular spheroids compared to adherent cells in the presence of cisplatin, which is indicative of reduced caspase activity [16]. To further confirm the role of Bcl-2 in the survival of multicellular spheroids treated with cisplatin, a Bcl-2 siRNA was used to demonstrate the contribution of Bcl-2 in these models [16]. The downregulation of Bcl-2 corresponded with the increased cleavage of pro-Caspase-3 and pro-Caspase-9. This concept was further supported by other investigators using the Bcl-2 inhibitor ABT-737 [17]. The treatment of SKOV3 multicellular spheroids with ABT-737 led to cisplatin sensitization as evidenced by an increase in Caspase-3 and Caspase-9 [16]. 

Albeit with limited sample numbers, another study compared Caspase-3 and Caspase-8 levels in normal ovary, benign mass and OvCa using flow cytometry caspase assay kits. Their results demonstrated low levels of Caspase-3 and Caspase-8 in the benign mass and malignant group compared to the normal ovary group [18]. A study by Yan et al. revealed increased levels of Caspase-8 via immunohistochemistry and correlated with increased patient survival as determined by Kaplan-Meyer analysis [19]. Similarly, Kim and colleagues showed that OvCa tumors had low levels of Caspase-8 and were associated with shorter overall survival compared to tumors from patients that had high levels of Caspase-8 [20]. 

### 2.2. Inhibitors of Apoptosis Proteins (IAP) in OvCa

The importance of IAP in promoting the pathology of OvCa became evident in multiple studies. X-linked inhibitor of apoptosis (XIAP) is one of the IAP family members, that regulates apoptosis (Figure 2), and does so by inhibiting Caspase-3, -7 and -9 [21]. Li et al. showed that cisplatin sensitive human OvCa lines (OV2008 and A2780s) treated with cisplatin displayed distinct cell morphology changes that accompany apoptosis such as decreased cell volume, chromatin condensation and nuclear fragmentation, and decreased protein levels of XIAP. These effects were not observed when cisplatin was added to their resistant counterparts (C13 and A2780cp) and the protein levels of XIAP remained unaffected [22]. The overexpression of XIAP by adenovirus sense cDNA in OV2008 cells failed to cause apoptotic nuclear fragmentation when treated with cisplatin. This confirmed the importance of XIAP in suppressing apoptosis and supporting chemoresistance [22]. An independent study by Mansouri and co-authors observed an increase in XIAP’s mRNA levels when cisplatin-resistant cells, C13, were treated with cisplatin [23]. The downregulation of XIAP by adenovirally delivered anti-sense cDNA induced apoptosis in cisplatin sensitive (OV2008) as well as in cisplatin-resistant (C13 *) malignant human ovarian surface epithelium (hOSE) cells carrying wild-type p53 as determined by assessing nuclear morphology (shrinkage, condensation and fragmentation) after Hoechst staining which aids in the assessment of apoptosis [24]. XIAP downregulation also re-sensitized resistant cancer cells to cisplatin treatment and led to an increase in apoptotic cell population [24]. However, this increase in apoptosis was not observed when XIAP was downregulated in p53-mutated cisplatin-resistant human ovarian epithelial adenocarcinoma cell line A2780. Combined, these studies highlighted the role of XIAP as an important determinant in cisplatin resistance and in regulating p53-mediated-apoptosis [24]. OV2008 cells, although studied extensively as a model for OvCa, have discrepancy in their origin [25]. However, regardless of their origin, disruption of the apoptotic pathway in these cells led to chemoresistance.

Interestingly, secretions from the cancer microenvironment can provide protection to cancer cells against apoptosis by modulating XIAP levels [26]. OVCAR3 cells, when pre-incubated with conditioned medium from cancer-associated mesenchymal cells (CA-MSCs), exhibited decreased pro-Caspase-3 and Caspase-7 cleavage, increased AKT phosphorylation and stabilization of XIAP protein levels in response to carboplatin treatment as determined by immunoblot analysis [26]. Such protection against apoptosis provided by conditioned media of CA-MSCs was abrogated when XIAP was depleted in OVCAR3 cells by siRNA and then treated with carboplatin. Apoptosis was restored in these cells as determined by Annexin-V- Fluorescein isothiocyanate (FITC) labeling and increased poly-ADP ribose polymerase (PARP) cleavage by immunoblot analysis [26]. PARP cleavage is a readout for increased protease activity and activation of cell death pathway. In another study, using anti-sense treatment of XIAP in *an* in vivo model utilizing A2780-cp cisplatin-resistant xenografts improved the overall survival of animals compared to scrambled anti-sense treated animals [27]. Histological analysis of the tumors also confirmed there were fewer viable cells in the anti-sense XIAP treated animals compared to the control tumors [27]. The investigators further demonstrated that when the cisplatin-resistant A2780-cp, cisplatin-sensitive A2780-s, and ES-2 OvCa cells were treated in vitro with the XIAP anti-sense construct, there was an increase in Caspase-3 as well as PARP cleavage compared to controls as determined by western blot analysis. These results indicate that XIAP conveys its apoptotic action through Caspase-3 and cleavage of PARP in these cells [27]. 

Survivin, another member of the IAP family, blocks apoptosis by inhibiting Caspase-3 and Caspase-7. The overexpression of survivin in OvCa cell lines IGROV-1 and OAW42 highlighted its role in influencing cell-sensitivity to taxanes (taxol and taxotere). Higher levels of survivin significantly decreased the susceptibility of IGROV-1 and OAW42 cells to taxanes including decreased apoptotic response as measured by terminal deoxynucleotidyl transferase dUTP nick end labeling (TUNEL) assay. However, it did not affect sensitivity of cells toward platinum compounds [28]. Additionally, survivin levels determined by IHC were inversely related to pathological or clinical complete response following taxol regimens in advanced OvCa patients indicating its role in tumor cell susceptibility to taxol [28]. A study by Chen et al. found that the positive expression of survivin as determined by IHC was associated with platinum resistance in OvCa patients [29]. Moreover, adenovirus mediated downregulation of survivin in cisplatin-resistant OvCa cells and A2780-cp, led to a marked increased in number of apoptotic cells as measured by FITC/PI-Annexin V staining as well as increase in levels of pro-Caspase-3 and cleaved Caspase-3 as measured by western blot analysis [30]. The depletion of survivin using anti-sense oligonucleotides in drug resistant OvCa cell line (COC1/DDP) led to an increase in apoptosis as determined by morphology and cell cycle analysis [31]. 

Another suppressor of apoptosis is Fas-associated death domain-like interleukin-1β-converting enzyme (FLICE)-like inhibitory protein (FLIP). It regulates cell surface receptor-mediated cell death by inhibiting activation of Caspase-8. In a study by Abedini and colleagues, cisplatin treatment decreased protein levels of FLIP and led to increased cleavage of Caspase-8 and Caspase-3 in cisplatin-sensitive cells (OV2008) but not in their cisplatin-resistant counterpart (C13 *) [32]. The overexpression of FLIP in cisplatin-sensitive cells attenuated the activation of caspases and apoptosis in response to cisplatin, while its downregulation by siRNA in cisplatin-resistant cells made them more responsive to cisplatin induced apoptosis. Collectively these studies emphasize the importance of FLIP as a chemotherapy resistance factor [32].

### 2.3. Ubiquitination Mediated Apoptosis in OvCa 

The ubiquitin-proteasome-system (UPS) is primarily responsible for regulating protein degradation via the proteasome by a process known as ubiquitination [33]. While there are several reviews outlining the specifics of ubiquitination [34,35], our focus will be on how ubiquitination steps are altered in OvCa to regulate apoptosis. In OvCa, an increase in aberrations in the UPS leads to more accumulated polyubiquitinated proteins despite an elevated level of proteasomal proteins. This was deemed indicative of an increase in cell proliferation and metabolic rate [35]. More specifically, the treatment of cultured ES-2 OvCa cells with a proteasome inhibitor for 24 h induced accumulation of endogenous cell checkpoint inhibitors p21 and p27, a 10-fold increase in Caspase-3 and increase in Annexin V staining, which is indicative of apoptosis [33]. In another study, the targeted inhibition of ubiquitin-specific protease 14 (USP14) using short hairpin RNA (shRNA) in SKOV3 cells led to a reduction in Bcl-XL levels as shown by western blot analysis which was concurrent in an increase in apoptosis as measured by Annexin V staining [36]. Further investigation revealed that USP14 interacts with the anti-apoptotic Bcl-XL as evidenced by co-immunoprecipitation and that the upregulation of USP14 disrupts the normal proteosomal degradation. This, in turn, would allow SKOV3 cells to avoid apoptosis [36]. Similarly, it was found that Cullin-really interesting new gene ubiquitin ligase 4 (CRL4), an E3 ubiquitin ligase made up of several components including viral protein R binding protein (VPRBP), cell division cycle protein 2 (CDT2), Damage-Specific DNA-Binding Protein 2 (DDB2) and Regulator of Cullins-1 (ROC1) are overexpressed OvCa tissues [37]. CRL4 is a member of the Cullin-RING ubiquitin ligases (CRL) family. MLN4924 is an NEDD8-activating Enzyme E1 (NAE) inhibitor that is needed to catalyze and activate CRL. MLN4924 treatment of OvCa cells leads to the inactivation of CRL/Skp Cullin F-box containing complex (SCF) E3 ligase and triggering of the DNA damage response. In turn, this induces apoptosis as measured by an increase in cleaved Caspase-3 and the accumulation of p21 and p27 using western blot analysis [37]. In summation, this work emphasizes the vulnerability of the UPS under stress in OvCa cells, some of which may be effective targets for OvCa treatment in the future.

Platinum chemotherapy agents such as cisplatin, which is a common first-line therapy for OvCa was also studied with relation to their effects on ubiquitination. MacKay et al. found that the E3 ubiquitin ligase HOIP/Ring Finger Protein 31 is a key regulator of cisplatin’s ability to destroy the cell’s genetic material, which is also known as genotoxicity [38]. Specifically, the depletion of HOIP sensitizes cisplatin-resistant A2780 cells and PEA-1 OvCa cells to cisplatin resulting in apoptosis as measured by Caspase-3 accumulation via western blot analysis [38]. Additionally, the cisplatin treatment of chemosensitive cells has been shown to induce the ubiquitination of Caspase-8/FADD-like IL-1 β-converting enzyme (FLICE)-like inhibitory proteins (FLIP) leading to their proteasomal degradation in a p53- and an E3 ligase called Itch, dependent manner [39]. The downregulation of FLIP by cisplatin treatment was shown to induce apoptosis in OV2008 and A2780 OvCa cell lines, as measured by Hoechst 33258 nuclear staining [39,40]. Selvendiran et al. demonstrated that treatment of cisplatin-resistant A2780 OvCa cells with the synthetic compound 3,5-bis(2-flurobenzylidene) piperidin-4-one (EF24) induced apoptosis by upregulating phosphatase and tensin homolog (PTEN) expression in vitro [41]. EF24 treatment elevated PTEN by blocking its ubiquitination and subsequent proteasomal degradation as well as apoptosis as measured by Caspase-3, Caspase-7, Caspase-8 cleavage and elevated FasL expression by western blot [41]. Similarly, cisplatin-resistant SKOV3 OvCa cells have elevated levels of p62 compared to cisplatin-sensitive cells [42]. The p62 protein binds ubiquitinated proteins and shuttles them towards autophagy, instead of endoplasmic reticulum stress induced apoptosis. This was measured by increased Caspase-4, and Caspase-3 protein levels which were glucose-regulated [42]. Therefore, upregulation of p62 allows the cisplatin-resistant cells to avoid apoptosis and survive cisplatin treatment in vitro [42]. Continued research into chemoresistance is necessary and the work presented here has shown a potential association between the UPS and platinum resistance in OvCa. 

## 3. Regulation of OvCa Apoptosis via Glycosylation and Glycan-Related Proteins 

Glycosylation is a post-translational modification where glycan molecules bind to a protein, lipid, or another carbohydrate to form a glyco-conjugate: a glycoprotein or proteoglycan, glycolipid, or oligosaccharide. Over half of all proteins in the human body are glycosylated [43]. These glycosylations are associated with multiple cellular processes including cell death, which has been extensively reviewed by Lichtenstein et al. and others [44,45]. Both N-linked and O-linked glycosylated proteins play a critical role in OvCa progression and chemoresistance [46,47]. Additionally, it has been shown that glycosylation can regulate the function of death receptors such as Fas via forming an extensive branching structure that would hinder the ability of ligands to bind and hence prevent the launching of the extrinsic apoptotic pathway [44,48]. 

### 3.1. O-Linked Glycosylation

The aberrant changes in O-linked glycosylation on the surface of OvCa cells has been shown to effect cell signaling pathways. The O-linked glycoprotein mucin 16 (CA125) has been the focus of many studies due to its use in monitoring OvCa treatment response [49,50,51]. Reinartz et al. have shown that silencing the CA125 gene with short hairpin RNA induces Caspase-3 dependent apoptosis in the H8, B8, MT, and SKOV3 OvCa cell lines as measured by Annexin V staining and Caspase-3 western blot analysis [52]. The expression of a single-chain variable fragments (scFvs) against surface CA-125 in OVCAR3 cells led to a downregulation in CA125 causing increased sensitivity to doxorubicin, cyclophosphamide, and etoposide chemotherapeutic reagents [53]. The increased sensitivity was concurrent with an increase in Caspase-3 and Caspase-9 protein levels compared to their levels in the OVCAR3 scFv control group [53].

The contribution of O-glycans in influencing OvCa cell apoptosis was evident in studies described by Queiroz et al. who demonstrated that p53 senses O-linked N-acetylglucosamine (O-GlcNAc) levels. Once these levels change, p53 can stabilize, translocate into the nucleus, and mediate transcription of the pro-apoptotic Bax, which then induces mitochondrial membrane permeabilization and apoptosis [54]. Interestingly, this stabilization effect was lost in OvCa cell lines that have mutated p53 [54]. In some instances, the expression of cell surface glycans on healthy cells can be used to promote the survival of nearby OvCa cells. Work by Connor and others demonstrated that peritoneal cells in the ovary could produce high levels of decoy receptor 3 (DcR3) which affects OvCa cells [55]. OvCa cells can bind to DcR3 based on their heparan sulfate proteoglycan expression pattern, altering their sensitivity to platinum-based chemotherapy and their propensity to enter apoptosis. A sialyltransferase involved in O-glycan elongation is sialyltransferase β-galactosamide α-2, 6-sialyltransterase 1 (ST6Gal1) which has been reviewed in OvCa by Wichert et al. [56]. The overexpression of ST6Gal1 in OVCAR4 OvCa cells that are exposed to cisplatin led to a reduction in Caspase-3 activation as measured by western blot analysis [57]. Additionally, targeting hypersialylated OvCa cells with α2, 6-linked sialic acid binding *Sambucus nigra* agglutinin (SNA) was shown to induce the apoptosis of IOSE-364 and SKOV3 OvCa cell lines [58]. SNA treatment was found to increase Bax and decrease Bcl-2 levels as well as increase levels of Caspase-3 and Caspase-9 as measured by western blot, to induce apoptosis [58]. 

### 3.2. N-Linked Glycosylation

N-Linked glycosylation has also been studied in OvCa. As aberrant glycosylation gains attention for promoting OvCa, different inhibitors of glycosylation are being developed and tested, such as resveratrol, which is a glucose transporter 1 (GLUT1) inhibitor [59]. Resveratrol has been shown to selectively kill OvCa cells through inhibition of the Akt/GLUT1 signaling pathway [59]. Further analysis of resveratrol treated OvCa cells, PA-1, SKOV3, and MDAH2774, showed that resveratrol disrupts the N-linked glycosylation of proteins resulting in endoplasmic stress-mediated apoptosis in the OvCa cell as measured by Annexin V staining [60]. 

## 4. Apoptosis Regulation by Galectins

Galectins are soluble proteins that bind glycans that contain β-galactosides. There are 15 galectins that have been identified and implicated in myriad cell functions, including growth, metastasis, and invasion [61]. Galectins -1, -3, -7 and -9 are the most extensively studied in OvCa. Galectin-1 is overexpressed in OvCa samples compared to normal ovary where it was not detected [62]. Elevated levels of galectin-1 were shown to predict poor prognosis as determined by immunohistochemistry, western blots and qPCR [62]. Using the cisplatin-resistant OvCa cell line A2780-cp, Zhang et al. were able to elucidate that when galectin-1 is knocked down in these cells via siRNA, the cells became more sensitive to cisplatin compared to their control counterparts [62]. This sensitivity was attributed to a decrease in key apoptotic protein levels including Bcl-2, and H-Ras, as determined by western blots and immunofluorescence [62]. Conversely, when Galectin-1 was over expressed in Hey cells, the expression of Bcl-2 increased and was seen primarily in the cytoplasmic fraction [62]. Galectin-1 was also implicated in targeting the Fas receptor in T-cells and breast carcinoma [63,64,65] but has not yet been reported in OvCa.

Galectin-3 has also been studied in OvCa models. Mirandola et al. demonstrated that when OvCa cell lines SKOV3, ID8 and primary cells from two patients were treated with a truncated form of Galectin-3 (which would block any endogenous galectin-3) there was a reduction in cell survival as determined by Annexin V staining, flow cytometry, cell proliferation (ViaLight cell proliferation and cytotoxicity Bioassay kit), motility and invasion [66]. Galectin-3 was additionally able to inhibit cell proliferation when OVCAR3 cells were treated with cisplatin as determined by a CCK-8 assay and colony formation assays [67]. Exogenous recombinant Galectin-3 was also capable of decreasing Caspase-3 activity in SKOV3 cells [68]. When OVCAR3 cells, which typically have low Galectin-3 levels, were forced to express Galectin-3, they exhibited increased migratory and invasive ability when measured using transwell assays [69]. Moreover, Galectin-3 overexpression in OVCAR3 cells led to a decrease in cell apoptosis as assessed by TUNEL assay post treatment with cisplatin compared to control [67]. To better assess the effects of Galectin-3 in OVCAR3 cells, the investigators measured protein levels of pro- and cleaved Caspase-3/9 as well as cytosolic and mitochondrial Cytochrome C in the presence or absence of cisplatin. When galectin-3 was increased, there was a downregulation in the cleaved forms of Caspase-3/9 as well as lower levels of cytosolic Cytochrome C and it was able to inhibit cisplatin induced apoptosis as determined by TUNEL assay [67]. Cai and colleagues evaluated tissue and serum samples from 102 patients and measured the levels of Galectin 3 and toll-like teceptor 4 (TLR4) and attempted to parallel their levels to tumor paclitaxel resistance. In chemoresistant patients, levels of Galectin-3 and TLR4 were elevated as determined by western blot and immunohistochemical analysis. The authors further investigated this relationship in vitro using SKOV3 and ES-2 cells, where they used Galectin-3 siRNA to decrease its expression, which led to a decrease in cell viability, as determined by a 3-(4,5-Dimethylthiazol-2-yl)-2,5-diphenyltetrazolium bromide (MTT) assay. Furthermore, when cells were exposed to exogenous galectin-3 for 30 min, cell viability was restored in a dose dependent manner [70]. The importance of TLR4 has been previously shown to enhance tumor survival, chemoresistance and proposed as a novel treatment target in chemoresistant OvCa [71]. It was suggested that paclitaxel acts as a TLR4 ligand which causes cells to become resistant and increase survival [72]. TLR4 plays a major role in inducing the expression of TRAIL, TRAIL receptors, and the neutralization of TRAIL ameliorated TLR4 induced apoptosis. Galectin-3, has been shown to augment the increased expression of TLR4 in OvCa cells treated with paclitaxel. This increased TLR4 was shown to be due to galectin-3 inhibiting Cav-1/TLR4 interaction which ultimately led to increased survival [70].

Galectin-7 was also identified as one of the new mitochondrial Bcl-2 interacting proteins in HeLa cells, a commonly used cervical cancer line via immunocapture and mass spectrometry. Their results suggest that the binding of galectin-7 to Bcl-2 may enhance the intrinsic apoptosis pathway [73]. Although these results were in cervical cancer cells, it would be of interest to determine whether this interaction plays a role in OvCa cells. This would be particularly interesting since galectin-7, has been linked to poor prognosis and resistance to paclitaxel [74,75]. Immunohistochemical analysis done on 63 formalin fixed OvCa patient samples by Kim et al. revealed that the increased expression of galectin-7 correlated with poor survival outcome [76]. Furthermore, using A2780-PAR OvCa cells they were able to show that silencing galectin-7 via siRNA led to a reduction in proliferation via MTT assays which are indicative of reduced metabolic activity and apoptosis [76]. 

Galactin-9 was also studied in the OvCa cell line, OVCAR3, where it was shown to inhibit cell proliferation by increasing reactive oxygen species (ROS) [77]. Treatment with galectin-9 led to a dose dependent decrease in the protein expression of Bcl-2. This decrease in Bcl-2 consequently led to an increased Bax/Bcl-2 ratio which induced Cytochrome C release, and an increase in Caspase-3 and Caspase-6 activity leading to apoptosis induction [77]. Collectively, the data published suggests a role of galectins in OvCa cell’s ability to control apoptosis. 

## 5. Epigenetic Modifications and Their Role in OvCa Apoptosis

There have been a plethora of studies investigating epigenetic modifications in cancer. Epigenetic modifications are changes in gene expression that do not cause a physical alteration in the DNA itself. Herein, we will discuss the roles that genetic regulation via miRNA, DNA methylation, and histone deacetylation in OvCa and apoptosis. 

### 5.1. miRNAs in OvCa Apoptosis

Small non-coding RNA (miRNA) have been shown to negatively regulate specific gene expression by either targeting the degradation of mRNA or preventing its translation into protein [78,79]. Several miRNAs regulate different aspects of the apoptotic pathway. The miRNAs mainly studied to determine their contributions to the maintenance, progression, and/or treatment resistance of OvCa are miRNA-25, -29c, -101, -128, -141, -182, -200a and -506 [78] but not all these miRNAs have been linked to apoptosis in OvCa and are outside of the scope of this review. Among the most studied miRNAs in OvCa that have a role in promoting the apoptotic process are miRNA-25 [80], miRNA-31 [81], and miRNA-21 [82]. In this section, we will focus on how these miRNAs function in the pathobiology of OvCa and how they can be manipulated to promote apoptosis. 

MicroRNA-25 is one of three mi-RNAs in a highly conserved cluster of micro-RNAs called miR-106b-25 [83]. miR-25 is involved in a variety of biological processes, including cell proliferation, migration, differentiation, and apoptosis [83]. Zhang et al. found that downregulation of miRNA-25 induces apoptosis by upregulation of the pro-apoptotic protein Bim in OVCAR3 and SKOV3 OvCa cell lines as measured by Annexin V staining [80].

As with most forms of cell regulation, one miRNA can have opposing functionality in different tumor types [84]. Previous work has shown that miR-31 expression can have opposing roles in different forms of cancer. For example, miR-31 can promote cell proliferation, invasion and in vivo tumorigenesis in colorectal and pancreatic cancer but inhibits proliferation in prostate or OvCa [84]. The diverse functionality of miR-31 may be due to its ability to interact with several major downstream pathways including PI3K/Akt and Ras/MAPK. Using OvCa cell lines with dysfunctional p53 activity, such as OVCAR8, OVCAR433, and SKOV3, it was found that the overexpression of miR-31 promoted apoptosis as measured by Caspase 3/7 activity, potentially through the cell cycle regulator, E2F2, which in turn increased p14^ARF^ transcription [81]. This increased level of apoptosis in cells over expressing miRNA-31 was not seen in cells with functional p53 such as HEY OvCa cells [81]. Previous work showed that miR-31 was also involved in promoting drug resistance through the tyrosine kinase receptor mesenchymal epithelial transition (MET) [85] and continues to be studied as a potential tumor suppressor in OvCa.

miR-21 has been the focus of several studies in OvCa. One demonstrated that the downregulation of miR-21 induced apoptosis in OVCAR3 cells as measured by Annexin V staining [86]. This study was expounded upon by Chan et al. who found that the inhibition of miRNA-21 induced apoptosis in the cisplatin-resistant OvCa cell line A2780-cp which was also assessed via Annexin V staining [82]. Liu and colleagues have shown that inhibition of miR-21 in A2780 or SKOV3 cells elevated levels of PTEN leading to decreased PI3K/Akt pathway activity and increased apoptosis as measured by Annexin V staining [87]. While there has been a moderate level of success with altering OvCa apoptosis in vitro, it would be interesting to see if these results can be duplicated in vivo.

Another miRNA studied in OvCa is miR-142-5p. miR-142-5p targets the XIAP protein function which leads to sensitizing OvCa cells such as SKOV3 and OVCAR3 to cisplatin treatments [88]. The TCGA revealed that patients with elevated levels of miR-142-5p had longer median progression-free survival compared to patients that had lower miR-142-5p levels [88] providing some clinical relevance. A list of some miRNAs that have been implicated in contributing to apoptosis of OvCa and corresponding citations are listed in Table 1.

### 5.2. DNA Methylation 

DNA methylation is an epigenetic alteration that adds methyl groups via an enzymatic process to specific promoter regions in the DNA leading to the suppression of that gene [117]. Genes that have CpG islands are especially susceptible to DNA methylation [118]. In cancer, the hypermethylation of CpG islands leading to the silencing of specific genes such as tumor suppressors has been well documented in the literature [119,120]. In OvCa, the hypermethylation of BRCA1, PTEN, Ras-association domain family 1 (RASSF1A), and altered regulation of LOT1, DAPK, TMS1/ASC, and PAR-4 as well as others, have been shown and reviewed by multiple investigators [120,121,122,123,124]. In addition to DNA hypermethylation, the enzymes that epigenetically modify chromatin such as H3K9 methyltransferase G9a, are also elevated in OvCa and correlated with poor outcomes and shorter survival [125]. Due to the importance of DNA methylation in cancer, drugs targeting the DNA methylation process have been developed. The most common are DNA methylation inhibitors such as 5-azacytidine (5-AZA) and decitabine (5-aza-2′-deoxycytidine). These were developed in the 1960s for treatment in hematological cancers. The food and drug administration (FDA) later approved these for use in myelodysplastic syndrome and have been investigated in solid tumors. However, due to their immunosuppressive effects, their use has been limited [126]. Regardless, platinum-resistant OvCa cells 2008/C13 were re-sensitized to carboplatin when pre-treated with azacytidine [127]. An increase in apoptotic cells was observed when 2008/c13 cells were sequentially treated with azacytidine and carboplatin as measured by TUNEL assay [127]. Furthermore, there was an induction in cleaved Caspase-3 and -8 by western blot analysis [127]. Another inhibitor, S2101, inhibits lysine-specific demethylase 1 (LSD1), a demethylase that specifically removes methyl groups from lysine residues of histone and non-histone proteins. LSD1 is overexpressed in various cancers, including OvCa [128]. Treatment of SKOV3 cells with S2101, led to a significant increase in apoptotic cells as measured by Annexin-V/ propidium iodide (PI) staining [129]. Additionally, inhibition of LSD1 in SKOV3 cells increased protein levels of Bax and decreased levels of Bcl-2 by western blot analysis, indicating that inhibition of LSD1 can induce apoptosis. 

TRAIL-mediated apoptosis, as discussed previously, involves the binding of TRAIL to its pro-apoptotic death receptors DR4 and DR5. Resistance to TRAIL-mediated apoptosis was observed in the OvCa cell lines A2780, A2780DR, and MZ-15, as quantified by DNA fragmentation analysis [130]. A2780 and A2780DR displayed a loss of DR4 expression, as measured by RT-PCR and western blot analysis, which is in contrast to the TRAIL-sensitive ovarian cancer cell lines MZ-26, ES-2, and CaOV-3 [130]. Horak et al. found that DR4 promoter hypermethylation correlated with lower levels of DR4 expression in MZ4, MZ37, and A2780 cells, as measured by RT-PCR, indicating epigenetic regulation of DR4 [131]. The treatment of A2780 cells with decitabine restored the expression of DR4 as measured by RT-PCR and re-sensitized them to TRAIL treatment leading to an increase in apoptotic cells as determined by APO-direct kit [131]. Similarly, transient transfection of full-length DR4 construct in A2780 cells re-sensitized them to TRAIL treatment and increased levels of apoptosis as measured by Annexin V assay [131]. Collectively, these studies suggest a role of epigenetic regulation of DR4 that leads to TRAIL mediated resistance in OvCa cells. 

Other pre-clinical studies have targeted the methyltransferases enhancer of zeste homolog 2 (EZH2) and BET. EZH2 is overexpressed in 50–85% of OvCa and correlates with high grade, advanced stage, and poor survival [132,133,134,135]. EZH2 inhibitors are in clinical trials for solid tumors such as lymphoma, but not yet for OvCa (NCT01897571). The importance of EZH2 in OvCa and apoptosis was indicated by Li et al., where they compared SKOV3 cells that had EZH2 silenced via short hairpin (sh)RNA and vehicle control cells. SKOV3 cells that had a lower level of EZH2 showed higher levels of Annexin V staining and increased cleaved Caspase-3 levels [136]. Additionally, their data suggests that this effect was likely mediated by methyltransferase H3K27Me3, as indicated with a decrease in protein levels of the enzyme via western blot analysis [136]. Another inhibitor, 3-Deazaneplanocin A (DZNep), which inhibits S-adenosylhomocysteine hydrolase is shown to deplete cellular levels of EZH2 in breast and colorectal cancer cells [137]. Treatment of A2780 OvCa cells with DZNep increased apoptosis as measured by Annexin-V and 7-AAD staining [138]. 

The use of DNA methylation inhibitors has shown some benefit in clinical chemoresistant OvCa. Matei and colleagues have shown that alterations in the DNA methylation process contribute to platinum resistance in OvCa [139]. In a clinical trial, decitabine was able to sensitize platinum-resistant patients [139]. A comprehensive list of relevant clinical trials using DNA methylation inhibitors as well as histone deacetylase (HDAC) inhibitors, as discussed below, in OvCa was summarized by Smith et al. [126]. Interestingly, the anti-diabetic drug metformin was implicated in repressing histone 3 lysine 27 tri-methylation (H3K27me3) via 5’ AMP-activated protein kinase (AMPK) phosphorylation. A phase II clinical trial was initiated and currently recruiting to assess the benefits of adding metformin to standard chemotherapy in non-diabetic OvCa patients (NCT02122185).

### 5.3. Histone Acetylation

Histone deacetylase enzymes are a group of enzymes that are known to silence genes via removing acetyl groups from histones as well as non-histone proteins [140]. The removal of the acetyl group alters the charge of the histones leading to a tighter bind to DNA, which then prevents gene transcription [140]. In contrast, histone acetyltransferases (HATs) can add an acetyl group.

In OvCa, HDAC6 is often elevated, leading to the inactivation of p53 apoptotic function. This was reversed when Bitler et al. used a small molecule HDAC6 inhibitor [141]. Conversely, the hypoacetylation of H3 and H4 was evident in a variety of OvCa cell lines [142]. Other HDAC inhibitors such as MS-275 have been shown to activate Caspase-8 and Caspase-3, as well as decrease Mcl-1 and XIAP protein levels in lymphoma and leukemia cells [143]. This suggests that HDAC inhibitors may directly affect apoptosis in cancer. Similarly, treatment with the BH3 mimetic AT-101 in combination with cisplatin in OvCa cell lines OVCAR3 and MDAH-2774 displayed a synergistic induction of apoptosis as determined by DNA fragmentation analysis [144]. 

Additionally, this treatment also led to increased inhibition of DNA methyltransferase (DNMT) and HDAC enzymatic activities as measured by Activity/Inhibition Assay kits [144]. However, the link between the inhibition of epigenetic modulators and the induction of apoptosis by AT-101 and cisplatin treatment needs further investigation. Treatment of the OvCa cell line SKOV3 with a pan-BH3 mimetic S1 led to an increase in apoptotic cells as measured by Hoechst staining [145]. Additionally, a decrease in levels of Bcl-2 and Mcl-1, and an increase in levels of cleaved Caspase-3, cleaved PARP, and cytoplasmic Cytochrome C was observed by western blot analysis [145]. This induction in apoptosis by S1 was partially attributed to SIRT3, a member of the sirtuin family of nicotinamide adenine dinucleotide (NAD)-dependent deacetylases. SKOV3 cells, when treated with S1, led to an increase in levels of SIRT3 as well as its mitochondrial localization, as observed by western blot and immunofluorescence analysis [145]. Furthermore, knockdown of SIRT3 in SKOV3 cells using siRNA decreased S1-mediated upregulation of Caspase-3 and Caspase-7 activity compared to cells treated with control siRNA highlighting SIRT3’s contribution towards S1-mediated apoptosis [145].

HDAC inhibitors, similar to DNA methylation inhibitors, have been used in clinical studies. However, a pan-HDAC inhibitor called vorinostat was tested in OvCa and did not show any benefit [146]. A few clinical trials have been conducted in OvCa using other epigenetic modifiers such as belinostat (NCT00301756). However, many patients had severe adverse reactions, and the studies were terminated. The use of epigenetic modifiers as single agents was quickly replaced with combinatorial treatments. In chemoresistant OvCa, a clinical trial was designed to test the effects of an HDAC inhibitor, entinostat, with avelumab. This clinical trial is still active but is not currently recruiting for phase II (NCT02915523). 

It is essential to note that epigenetic therapeutic modalities gained additional interest after the discovery that some therapies can trigger an immune response. With the advent of immune therapies and their role in cancer, it would be interesting to determine whether combinatorial treatment of epigenetic drugs and immunotherapy has any added benefit in OvCa treatment.

## 6. Clinical Use of Drugs Targeting Apoptosis in Ovarian Cancer

There are a few drugs and inhibitors that have been shown to induce apoptosis that are in clinical trials. We speculate that combining these with current chemotherapy can help overcome chemoresistance in OvCa and are summarized in Table 2. 

Clinical and pre-clinical studies are underway for using glycolysis inhibitors such as dicumarol. Dicumarol is an FDA approved naturally occurring anticoagulant that depletes vitamin K but can also inhibit pyruvate dehydrogenase kinase 1 (PDK1) activity in a cell free system [147]. In cells, PDK1 has been shown to be overactive in OvCa cells such as SKOV3 and A2780 and inhibited with dicumarol treatment as shown by flow cytometry via measuring ROS and mitochondrial membrane potential [147]. Annexin IV/PI staining also revealed that dicumarol treatment in A2780 led to increased levels of apoptosis and reduced MTT levels, which indicated reduced viability [147]. In vivo, SKOV3 tumor-bearing mice treated with dicumarol showed a decrease in tumor volume compared to vehicle treated mice. Additionally, levels of cleaved Caspase-3 and cleaved PARP were elevated in tumors hosted by animals treated with dicumarol when compared to tumors from the vehicle group [147]. This suggests that dicumarol may be conveying its action via increasing apoptosis in these cells and warrants investigation in OvCa. 

Another class of drugs being developed for targeting apoptosis are epothilones. Epothilone B is a member of a drug family that targets microtubules [148]. It conveys its action via binding to the α/β tubulin subunit and prevents microtubule detachment from centrosomes and mediates cell apoptosis [148]. Rogalska et al illustrated that epothilone B mediated apoptosis in OvCa OV90 cells occurred via the extrinsic pathway mediated by TRAIL and Caspase-8 [148]. Epothilone B was in clinical trials for multiple cancers, one of which was OvCa (NCT00035100).

Interestingly, the common antidiabetic medication, metformin, has also been investigated with regards to its effects on OvCa. Metformin induced dysfunctional changes in the mitochondria were described by Ma et al in OVCAR3, SKOV3 and HO8910 OvCa cell lines. These dysfunctions were shown to occur by an accumulation of ROS and activation of the ASK1 pathway in the presence of low glucose levels in the media. A mechanism of action by which metformin works is through inhibiting the mammalian target of rapamycin (mTOR) pathway circumventing p53-induced apoptosis [149]. These results and others led to the initiation of a clinical trial in OvCa in combination with cisplatin/carboplatin therapy (NCT02312661). Once completed it may provide additional support for the role of metformin in regulating apoptosis in OvCa cells.

Pharmacological inhibitors of IAPs, frequently referred to as Smac-mimetics (SM), have been in development, and some are being tested in clinical trials for cancer treatment. The challenges of using these IAP inhibitors were reviewed by Fulda et al [150]. DEBIO 1143 is one example studied in an in vitro model utilizing OvCa cell lines that are carboplatin sensitive such as A2780S, SKOV3 and IGROV-1 as well as the carboplatin-resistant cell lines (A2780R, SKOV-3 and EFO-21). Results demonstrated that DEBIO 1143 decreased levels of cIAP1 via western blot analysis [151]. Mice harboring SKOV3 tumors subcutaneously or interperitoneally treated with DEBIO 1143 alone or in combination with carboplatin were assessed for tumor burden [151]. Subcutaneous tumors regressed with DEBIO 1143. A survival study was performed with mice that were intraperitoneally injected with tumor cells and then treated with vehicle, DEBIO 1143, carboplatin, or DEBIO 1143 + carboplatin [151]. About 60% of mice survived in the combinatorial treatment arm compared to 20% in carboplatin alone and ~10% in the vehicle or DEBIO 1143 treatments [151]. DEBIO was shown to act via restoration caspase activity and regulating NFκB signaling and TNF-α. DEBIO 1143 was assessed for safety and pharmacodynamics in 2015 [152], and consequently used in clinical trials for OvCa and other solid tumors (NCT01930292). Currently, a phase I study is recruiting for patients with non-small cell lung cancer with avelumab with potential to be expanded to other solid tumors (NCT03270176). A trial recently started testing DEBIO 1143 in colorectal and pancreatic cancer patients in combination with pembrolizumab (NCT03871959). It remains to be seen whether DEBIO 1143 results would translate favorably to humans with solid tumors such as OvCa. 

LCL161 is a small antagonist of IAPs that has been studied in multiple cancer types such as B-cell lymphoma and has been shown to inhibit IAPs. The decrease in IAP levels was shown to be dose-dependent and enhanced the effects of cytotoxic chemotherapy [153]. LCL-161 is being tested in combination with the OvCa drug topotecan. The clinical trial is now in phase II and recruiting patients (NCT02649673) and is expected to complete recruitment in 2019. 

Another SM studied in OvCa is birinapant. It is specifically designed to target IAPs, cIAP1 and cIAP2 and activate Caspase-3 [154]. These results led to the initiation of a phase I dose escalation study in OvCa (NCT01940172). After 11 patients were treated, accrual was terminated due to lack of clinical benefit. However, further analysis is under way to determine the mechanism of action of birinapant and assess whether synergistic combinatorial treatments would be of benefit [154]. 

A small-molecule inhibitor called ABT737 targets the Bcl-2/Bcl-XL complex and induces apoptosis in the OvCa cell line SKOV3 and the cisplatin-resistant SKOV3/DDP via enhancing the activity of pro-apoptotic regulators such as caspases [17]. ABT737 was tested in animal models, including NSLC, where it improved survival and resulted in tumor regression. However, ABT-737 bioavailability was limited. The second generation, ABT-263, also known as navitoclax showed promise in hematological cancers in clinical trials but was halted due to increased incidence of thrombocytopenia which was attributed to the platelets’ need for Bcl-XL to survive [155]. A more recent small-molecule Bcl-2 inhibitor, venclexta (ABT-199), which has been approved for acute myeloid leukemia and chronic lymphocytic leukemia, is being investigated for the treatment of multiple cancers including some solid tumors. Although there are no specific clinical trials for OvCa, it would be of interest to determine whether this improved small molecule would benefit OvCa patients. However, a post-trial observational study on OvCa was performed and aimed to quantify the exvivo induction of apoptosis by ABT-737 with platinum exposed samples. This was done to determine whether there are advantages for using combinatorial treatments compared to single agents (NCT01440504), but has not gone any further in OvCa clinical trials. Another drug targeting Bcl-2 is geneta (G-3139), which modulates apoptosis in several cancers such as melanoma and lymphoma. Geneta reached phase III clinical trials but failed to show any benefit in progression-free survival, response rate and disease control rate (NCT00070343) and hence was not tested in other cancers [156]. 

As previously mentioned, the p53 pathway becomes dysregulated in most cancers, including OvCa, which prevents its anti-tumor function. p53 mutations are correlated with shortened progression-free interim and decreased overall survival in general and specifically in OvCa [157,158]. For a long time, p53 was considered undruggable, but recently, targeting p53 via small molecules or genetic restoration has been showing promise in pre-clinical settings. The first candidate drug targeting p53 reaching clinical trials was APR-246, which is also known as PRIMA-1MET [159]. Its mechanism of action was shown to reactivate mutant p53 in cancer cells by promoting the correct folding, which then triggers apoptosis [159,160,161,162]. APR-246 was tested in phase Ib in OvCa patients and the results showed that it was well tolerated with chemotherapy and is being evaluated in a phase II study (NCT03268382). Recently, a small molecule targeting p53, called kevetrin, has demonstrated the potential of becoming a breakthrough cancer treatment by activating p53. Kevetrin induces apoptosis by both activations of wild-type p53 and by inducing apoptosis in mutant p53 cells by degradation of oncogenic mutant p53. Presently, kevetrin is in phase II clinical trials for OvCa (NCT03042702).

An interesting drug in phase II clinical trials for solid tumors including OvCa, is the DPX-Survivac vaccine (NCT03836352). This is a synthetic survivin peptide attached to an adjuvant which creates a depot that releases survivin. This allows for a prolonged exposure of survivin to the immune system that provokes a strong immune response against cells that make increased levels of survivin. Since cancer cells have elevated levels of survivin, an immune response is mounted leading to a decrease in tumor cell proliferation and an increase in apoptosis. An additional phase II trial that is currently recruiting (NCT03029403) will test a combinatorial treatment of DPX-Survivac, Keytruda and Cytoxan in patients with advanced stage OvCa, fallopian tube cancer, or primary peritoneal cancer.

Another druggable target was aalectin-1. The importance of galectin-1 in OvCa prompted a group of investigators to study the effects of a small molecule galectin-1 inhibitor called OTX008 [163]. In A2780-1A9 OvCa cells, OTX008 was shown to decrease the expression of galectin-1 and decreased ERK1/2 and AKT-dependent survival pathways, as well as induced cell cycle arrest in the G2/M phase via CDK1. Moreover, when tested in vivo, OTX008 treated animals displayed an inhibition of A2780-1A9 xenograft growth which was associated with decreased levels of galectin-1 and Ki67 positive cells [163]. A phase I clinical trial was completed with OTX008 in solid tumors (NCT01724320) and it was well tolerated by patients. However, there is no indication that the compound was developed further or progressed to phase II clinical trials. 

One of the newer drugs in this space is CPI-613, which is a member of lipoate derivatives that inactivate the Tricarboxylic acid (TCA) cycle by the phosphorylation of tumor cell pyruvate dehydrogenase via PDK1-4. This inactivation leads to the shutdown of mitochondrial function and activation of death pathways [164]. This mechanism of action works because one of the characteristics of many cancers is the presence of elevated metabolism, particularly to withstand the hypoxic environment and anabolic demands of a tumor environment [165]. Although initial research showed that glycolysis is the main pathway for ATP generation in cancer cells while the oxidative phosphorylation pathway was impaired (Warburg-effect) [166], recent reports have shown that both pathways are elevated in cancer cells [167]. Additionally, some tumors were found to contain two subpopulations of cells, one utilizing the glucose-dependent metabolism (Warburg-effect) and the second population exhibited increased utilization of lactate in the TCA cycle [168,169]. Cancer cells that are quiescent and in avascular regions tend to have increased mitochondrial activity using the oxidative phosphorylation pathway. This pathway functions optimally at 0.5% oxygen levels and renders the cell to become quiescent and less proliferative, which leads to chemoresistance. The hypoxic environment is what drives the mitochondria to switch ATP production pathways and hence, plays a major role in drug resistance [170]. CP-613 is also able to inactivate α-ketoglutarate dehydrogenase (KGDH) pathway in non-small cell lung carcinoma [171]. The ability of CP-613 to target the alternative metabolic pathways is a way to render cancer cells more sensitive to chemotherapy and apoptosis. In tumor cells, CPI-613 induces an increase in ROS, causing a disruption in redox balance and tumor cell death and sparing normal cells [171]. CPI-613 has been in clinical trials for pancreatic cancer and is currently recruiting for phase III (NCT03504423). CPI-613 is also in clinical trials in hematological malignancies such as lymphoma and leukemia (NCT03793140). Even though it has not been cleared for a clinical trial in OvCa, it would be interesting to determine whether similar results would be seen in OvCa. 

## 7. Conclusions

Whether it be an indirect or direct inhibition of extrinsic or intrinsic signaling that promotes apoptosis, the end-point is the same. The tumor cells have gained a survival advantage which often equates to a more resistant phenotype. These apoptotic resistant cells likely contribute to the high recurrence rates of chemoresistant disease. Developing some type of companion diagnostic to help direct anti-cancer therapies designed to disrupt specific cell signaling pathways or override epigenetic modifiers that inhibit apoptosis would be beneficial. However, given the inherent and often acquired heterogeneity of OvCa, it is likely that some cells are not reliant on apoptosis but other forms of programmed cell death. Identifying key modulators of the primary pathways enabling OvCa cells to evade apoptosis or the other forms of cell death could also serve as the basis for the development of new alternative or complementary treatment modalities. 

## Figures and Tables

**Table 1:** miRNAs reported in OvCa with a possible role in apoptosis.

microRNA (miRNA)	Function	Methods Used	Citation
miRNA-15a	Bmi-1 is elevated in OvCa cells and tissue samples There is an inverse correlation between miRNA15a and Bmi-1 over expressing miRNA-15a down regulates Bmi-1 protein in OvCa reducing cell proliferation	qPCR, western blot analysis, reporter assay	Bhattacharya et al., 2009 [89]
miRNA-16	miRNA-16 down regulates Bmi-1 (Battacharya, 2009) and Bcl-2 (Cimmino, 2005) protein expression	Western blot analysis, TUNEL	Bhattacharya et al., 2009 [89], Cimmino et al., 2005 [90]
miRNA-18a	Overexpression of miRNA-18a in OvCa cell lines decreases levels of TRIAP1, IPMK, and cleaved Caspase 3 and increases apoptosis in vivo	Western blot analysis, TUNEL	Liu et al., 2017 [91]
miRNA-21	Inhibition of miRNA 21 resulted in reduced pAKT and upregulated PTEN (Liu, 2019) and c-IAP2 (Chan, 2014) which interferes, with caspase activation in human OvCa cell lines.	Reporter assay, western blot analysis, qPCR, Annexin V	Chan et al., 2014 [82], Lou et al., 2011 [86], Liu et al., 2019 [87]
miRNA-25	Knockdown of miRNA-25 in OvCa cell lines decreases Bim and Bcl2 levels, increases, Bax, cleaved Caspase-3 levels and apoptosis	Annexin V, western blot analysis	Zhang et al., 2012 [80], Sarkozy et al., 2018 [83]
miRNA-31	In p53 deficient OvCa cell lines, miRNA-31 can induce p53-mediated apoptosis indirectly by targeting *E2F2*, *MIR31*, and *CDKN2A* genes.	qPCR, molecular profiling	Creighton et al., 2010 [81]
miRNA-34	OvCa cell lines transfected with miRNA-34 mimics increase levels of Bax, while decreasing levels of Bcl-2 leading to increased apoptosis	Annexin V, western blot analysis	Jia et al., 2019 [92]
miRNA-93-5P	Overexpression of miRNA-93-5P in OvCa cell lines decreases levels of Bcl/xl, cleaved PARP, and increases levels of p53 and apoptosis.	Annexin V, western blot analysis	Chen et al., 2015 [93]
miRNA-106a	Inhibition of miR-106a enhanced the sensitivity of the OvCa cells to chemotherapy and increased apoptosis. Increasing miRNA-106a decreased PDCD4 levels. PDCD4 silencing led to a decrease in cleaved pro-Caspasse-3 and -9.	Flow Cytometry, western blot analysis	Rao et al 2013 [94], Li et al, 2014 [95]
miRNA-124	Overexpression levels of miRNA-124 increases apoptosis and decreased levels of PDCD6 in OvCa cell lines	Annexin V, qPCR	Yuan et al., 2017 [96]
miRNA-130a	miRNA-130a downregulates XIAP in human A2780 cells.	qPCR, western blot analysis, flow cytometric analysis, reporter assay, Annexin V	Zhang et al., 2013 [97]
miRNA-135a	Transfection with an miRNA-135a mimic increases Caspase-3 activity and p53 levels and decreases Bcl-2 levels in OvCa cell lines.	Western blot, Caspase-3 activity assay, Annexin V	Tang et al., 2014 [98]
miRNA-137	miR-137 knockout by CRISPR/Cas9 increases XIAP levels and inhibits apoptosis in OvCa cell lines	TUNEL, DAPI, western blot analysis	Li et al., 2017 [99]
miR-142-5p	miRNA-142-5p inhibits XIAP in human OvCa cell lines. miR-142-5p targeted anti-apoptotic genes (*Birc3*, *Bcl2*, *Bcl2L2* and *Mcl1*)	Dual luciferase assay, western blot analysis, flow cytometric analysis	Li et al., 2019 [88], Su et al. 2019 [100]
miR-147b	Elevated miRNA-147b results in increased Bak1 and Bax levels and reduces levels of Bcl-2 and Bcl-xl in SKOV3 cells.	Molecular profiling, western blot analysis, Caspase-3 and 7 activity, mitochondrial potential	Kleemann et al., 2017 [101]
miRNA-149	Downregulation of miRNA-149 decreases apoptosis in OvCa cells pre-treated with paclitaxel. Downregulation of miRNA-149 decreases Bax mRNA and protein and increases Bcl-2 mRNA and protein expression inhibits XIAP expression in human OvCa cells. Overexpression of miRNA-149 decreases XIAP mRNA and protein expression and increases apoptosis in OvCa cell lines.	Annexin V, qPCR, western blot analysis	Sun et al., 2018 [102], Zhan et al., 2015 [103]
miRNA-152	Overexpression of miRNA-152 decreased DNMT1 and increased apoptosis and in OvCa cell lines.	Annexin V, western blot analysis, qPCR	Xiang et al., 2014 [104]
miRNA-181a	Overexpression of miRNA-181a decreased apoptosis in OvCa cells	Annexin V	Li et al., 2016 [105]
miRNA-193a and miR-193b	Overexpression of miRNA-193a or miRNA-193b increases activity of Caspase-3 and -7 in OvCa cells. miRNA-193a also decreases levels of anti-apoptotic factor MCL1 in OvCa cells	Caspase-3 and -7 activity, western blot analysis	Nakano et al., 2013 [106]
miRNA-195-5p	Overexpression of miRNA-195-5p increases apoptosis in OvCa cells and in a in vivo model.	Annexin V, TUNEL	Dai et al., 2019 [107]
miRNA-221	Inhibiting miRNA-221 increases APAF1 and apoptosis in OvCa cells	Annexin V, western blot analysis, Hoechst 33342 staining	Li et al., 2017 [108]
miRNA-338-3p	miRNA-338-3p induces apoptosis by binding to long non-coding RNA LINC00460.	Luciferase reporter assay, western blot analysis, flow cytometric assay	Liu et al., 2018 [109]
miRNA-493-3p	Overexpression of miRNA-493-3p in OvCa cell lines increased Bak levels, release of cytochrome C, cleavage of Caspase-3 and PARP and decreased Bcl-XL levels	Western blot analysis, free cytochrome C staining, Annexin V	Kleeman et al., 2019 [110]
miRNA-614	Overexpression of miRNA-614 decreases level of Bad and increases apoptosis in OvCa cells.	Annexin V, western blot analysis	Zhang et al., 2018 [111]
miRNA-630	Silencing of miRNA-630 in OvCa cell lines increases cleaved Caspase-3, PTEN levels and apoptosis.	Annexin V, western blot analysis	Eoh et al., 2018 [112], Zou et al., 2015 [113]
miRNA-718	Overexpression of miRNA-718 in OvCa cell lines decreases VEGF levels and increases apoptosis. This is reversed when VEGF is restored	Annexin V, western blot analysis	Leng et al., 2014 [114]
miRNA-744-5p	Increased expression of miRNA-744-5p increased levels of cleaved Caspase-3, and PARP and decreased levels of Bcl2 in OvCa cell lines.	AnnexinV, western blot analysis, Caspase-3 and 7 activity, mitochondrial membrane potential	Kleemann et al., 2018 [115]
miRNA-1284	Inhibiting miRNA-1284 results in increased Bcl-2 levels and decreased Bax, and cleaved Caspase-3 levels in OvCa cell line. An miRNA-1284 mimic increases apoptosis	Annexin V, western blot analysis	Pan et al., 2016 [116]

TRIAP1: TP53 Regulated inhibitor Of apoptosis 1, MIR31: miRN-31, XIAP: X-linked inhibitor of apoptosis., IPMK: Inositol polyphosphate multikinase, CDKN2A: Cyclin dependent kinase inhibitor 2A, DNMT1: DNA methyltransferase 1, PTEN: Phosphatase and tensin homolog, PARP: poly ADP ribose polymerase, VEGF: Vascular endothelial growth factor, c- IAP2: Inhibitor of Apoptosis-2, PDCD4: Programmed cell death receptor 4, APAF1: Protein activating factor 1, E2F2: E2F transcription factor 2, PDCD6: Programmed cell death receptor 6.

**Table 2:** Summary of drugs with apoptosis related targets and clinical trial information.

Drug Treatment	Suspected Target Related to Apoptosis	Last Reported Phase	Cancer Type	NCT
Epothilone B	TRAIL and Caspase-8	Phase II	Recurrent ovarian	NCT00035100
Epothilone B versus Doxorubicin	TRAIL and Caspase-8	Phase III	Ovarian, Primary Fallopian, or Peritoneal Cancer	NCT00262990
Epothilone B + Omeprazole + Midalzolam	TRAIL and Caspase-8	Phase I	Advanced malignancies	NCT00420615
Metformin + Paclitaxel + Carboplatin	mTOR pathway circumventing p53-induced	Phase II	Advanced stage ovarian carcinoma	NCT02437812
DEBIO 1143+ Carboplatin + Paclitaxel	cIAP1	Phase II	Epithelial ovarian cancer	NCT01930292, NCT03270176
DEBIO 1143+ Avelumab	cIAP1	Phase I	Advanced solid malignancies	NCT03270176
LCL161	XIAP, cIAP1 and cIAP2	Phase II	Solid tumors	NCT02649673
Birinapant	IAPs, cIAP1 and cIAP2 and activate Caspase-3	Phase I/II	Solid tumors	NCT01940172
ABT 737/ABT 263 (navitoclax)	Bcl-2/Bcl-XL	Ex vivo study	Ovarian tumors	NCT01440504
Venclexta (ABT-199)	Bcl-2 inhibitor	Approved	Chronic lymphocytic leukemia	-
PRIMA-1^MET^ (APR-246)	p53	Phase II	High-grade serous ovarian cancer, high grade serious ovarian cancer (Platinum-Resistant)	NCT02098343, NCT03268382 (biomarker ID)
Kevetrin	p53	Phase II	Ovarian cancers	NCT03042702
DPX-Survivac	Survivin peptide attached to an adjuvant	Phase II	Ovarian cancers	NCT03836352, NCT03029403
OTX008	Galectin-1	Phase I	Solid tumors	NCT01724320
CPI-613	Alternative metabolic pathways	Phase III	Solid tumors	NCT03504423 (pancreatic)
CPI-613	Alternative metabolic pathways	Phase II	Lymphoma/Leukemia	NCT03793140

TRAIL: TNF-related apoptosis-inducing ligand, XIAP: X-Linked inhibitor of apoptosis, IAP1: Inhibitor of apoptosis protein 1, cIAP1: C-inhibitor of apoptosis protein, mTOR: Mammalian target of rapamycin, cIAP2: Inhibitor of apoptosis protein 2.

## Abbreviations

AMPK	5′ AMP-activated protein kinase
CA125	Cancer antigen 125, mucin 16
CA-MSCs	Cancer-associated mesenchymal cells
CDT2	Cell division cycle protein 2
CRL	Cullin-RING ubiquitin ligases
CRL4	Cullin-really interesting new gene ubiquitin ligase 4
DcR3	Decoy Receptor 3
DDB2	Damage Specific DNA Binding Protein 2
DISC	Death-inducing signal complex
DNMT	DNA methyltransferase
EF24	3,5-bis(2-flurobenzylidene) piperidin-4-one
FADD	Fas-associated death domain protein
FLIP	IL-1 β-converting enzyme (FLICE)-like inhibitory proteins (FLIP)
FasL	Fas ligand
FDA	Food and drug administration
FLICE	Fas-associated death domain-like interleukin-1β-converting enzyme
FLIP	FLICE-like inhibitory protein
FITC	Fluorescein isothiocyanate
GLUT1	Glucose transporter 1
hOSE	Human ovarian surface epithelium
IAP	Inhibitor of apoptosis
KGDH	α-Ketoglutarate dehydrogenase
MAPK	Mitogen-activated protein kinase
miRNA	Small non-coding RNA
MTT	3-(4,5-Dimethylthiazol-2-yl)-2,5-diphenyltetrazolium bromide
mTOR	Mammalian target of rapamycin
NAD	Nicotinamide adenine dinucleotide
NAE	NEDD8-activating Enzyme E1
NF-κB	Nuclear factor κB
O-GlcNAc	O-linked N-acetylglucosamine
OvCa	Ovarian cancer
PARP	Poly-ADP ribose polymerase
PDK1	Pyruvate dehydrogenase kinase 1
PI	Propidium iodide
PTEN	Phosphatase and tensin homolog
ROC1	Regulator of Cullins-1
ROS	Reactive oxygen species
SCF	CRL/Skp Cullin F-box containing complex
scFvs	Single chain variable fragments
shRNA	Short hairpin RNA
SM	Smac-mimetics
SNA	*Sambucus nigra* agglutinin
ST6Gal1	Sialyltransferase β-galactosamide α-2, 6-sialyltransterase 1
TCA	Tricarboxylic acid
TLR4	Toll-Like Receptor 4
TNF	Tumor necrosis factor
TRAIL	TNF-related apoptosis-inducing ligand
TUNEL	Terminal deoxynucleotidyl transferase dUTP nick end labeling
UPS	Ubiquitin-proteasome-system
UPS14	Ubiquitin-specific protease 14
VPRBP	Viral protein R binding protein
